# Proximity labelling reveals effects of disease-causing mutation on the DNAJC5/cysteine string protein α interactome

**DOI:** 10.1042/BCJ20230319

**Published:** 2024-01-29

**Authors:** Eleanor Barker, Amy E. Milburn, Nordine Helassa, Dean E. Hammond, Natalia Sanchez-Soriano, Alan Morgan, Jeff W. Barclay

**Affiliations:** Department of Biochemistry, Cell and Systems Biology, ISMIB, University of Liverpool, Liverpool, U.K.

**Keywords:** exocytosis, neurodegeneration, neuronal ceroid lipofuscinosis, PC12 cell, SNARE proteins, STXBP1

## Abstract

Cysteine string protein α (CSPα), also known as DNAJC5, is a member of the DnaJ/Hsp40 family of co-chaperones. The name derives from a cysteine-rich domain, palmitoylation of which enables localisation to intracellular membranes, notably neuronal synaptic vesicles. Mutations in the *DNAJC5* gene that encodes CSPα cause autosomal dominant, adult-onset neuronal ceroid lipofuscinosis (ANCL), a rare neurodegenerative disease. As null mutations in CSP-encoding genes in flies, worms and mice similarly result in neurodegeneration, CSP is evidently an evolutionarily conserved neuroprotective protein. However, the client proteins that CSP chaperones to prevent neurodegeneration remain unclear. Traditional methods for identifying protein–protein interactions, such as yeast 2-hybrid and affinity purification approaches, are poorly suited to CSP due to its requirement for membrane anchoring and its tendency to aggregate after cell lysis. Therefore, we employed proximity labelling, which enables the identification of interacting proteins *in situ* in living cells via biotinylation. Neuroendocrine PC12 cell lines stably expressing wild type or L115R ANCL mutant CSP constructs fused to miniTurbo were generated; then the biotinylated proteomes were analysed by liquid chromatography–mass spectrometry and validated by western blotting. This confirmed several known CSP-interacting proteins, such as Hsc70 and SNAP-25, but also revealed novel binding proteins, including STXBP1/Munc18-1. Interestingly, some protein interactions (such as Hsc70) were unaffected by the L115R mutation, whereas others (including SNAP-25 and STXBP1/Munc18-1) were inhibited. These results define the CSP interactome in a neuronal model cell line and reveal interactions that are affected by ANCL mutation and hence may contribute to the neurodegeneration seen in patients.

## Introduction

Cysteine string protein (CSP) was initially discovered in a search for *Drosophila* brain-enriched proteins, and named after a cluster of cysteine residues within the central region of the protein [1]. Subsequently, CSP homologues were identified in various multicellular organisms. Although the genomes of invertebrates such as *Drosophila* and *Caenorhabditis elegans* encode only a single CSP homologue, mammals contain three genes: *DNAJC5*, *DNAJC5B* and *DNAJC5G*, encoding CSPα, β and γ, respectively. DNAJC5/CSPα is by far the best studied and is thought to be the major isoform expressed in the brain (but see [2]). CSPα is enriched in neurons, where it mainly localises to synaptic vesicles [3]. Other secretory cells, such as adrenal chromaffin [4] and pancreatic beta cells [5], similarly express high levels of CSPα that mainly localise to regulated secretory vesicles. In contrast, CSPα localises to the plasma membrane in adipocytes [6] and to endosomal/lysosomal organelles in fibroblasts [7] and A549 cells [8]. Trafficking of CSP to post-Golgi membranes requires *S*-palmitoylation of cysteine residues within the cysteine string domain [9–11].

The localisation of CSP to synaptic and secretory vesicles suggests a functional role in regulated exocytosis. Indeed, early studies demonstrated defects in synaptic transmission in *Drosophila* csp mutants [12,13]. Subsequently, CSPα was shown to affect both early and late stages of calcium-dependent exocytosis in various secretory cells, including adrenal chromaffin cells [14,15], pancreatic beta cells [5,16] and PC12 cells [17,18]. The key neuroprotective role of CSP was revealed by studies in model organisms, where null mutations in CSP orthologues result in neurodegeneration in flies [12], mice [19] and worms [20]. Mutations in human DNAJC5/CSPα cause autosomal dominant, adult-onset neuronal ceroid lipofuscinosis (ANCL), a rare disease characterised by neurodegeneration, sensorimotor dysfunction and early death [21]. Four ANCL-causing mutations have been described to date, but all cluster around the cysteine string domain. The first discovered and most common recurrent variants are a Leu115Arg substitution (L115R) and a Leu116 single amino-acid deletion (L116Δ) [22–25]. Recently, two further ANCL-associated variants have been reported: a duplication of residues Cys124–Cys133 [26] and a Cys128Tyr substitution [27]. It is generally accepted that the L115R and L116Δ mutations induce oligomerisation/aggregation of CSP, although whether this requires palmitoylation of the cysteine string has been contentious [28–31]. It was recently reported that mutant CSP aggregation is caused by ectopic misloading of iron–sulfur (Fe–S) clusters by iron–sulfur cluster assembly enzyme (ISCU), which leads to co-aggregation with wild-type CSP, thereby providing a molecular explanation for the dominant nature of ANCL mutations [29].

CSP contains a DnaJ domain, which enables it to bind to and activate its co-chaperone Hsc70 [32,33]. Hence, it has long been accepted that a major cellular function of CSP is to maintain the correct folding of client proteins at neuronal synapses [34]. Compelling evidence suggests that the synaptic SNARE protein, SNAP-25, is a functionally important substrate, as it misfolds and is degraded by the proteasome in CSP knockout mice, leading to neurodegeneration [35,36]. In addition, a variety of different proteins that directly or indirectly modulate synaptic vesicle exo/endocytosis have been shown to bind to CSP and so represent further potential client proteins. These include the two other synaptic SNARE proteins, VAMP2 [37] and syntaxin-1 [15,38,39], the exocytotic calcium sensor synaptotagmin [40,41], the endocytic protein dynamin-1 [42], G-protein subunits [43,44], 14-3-3 proteins [45], calcium channels [37,46] and potassium channels [47]. In the last few years, a different neuroprotective role has been proposed for CSP: as a downstream mediator in misfolding-associated protein secretion (MAPS) [48–51]. In this process, misfolded proteins, such as α-synuclein and tau, are recruited onto the endoplasmic reticulum by USP-19 and deubiquitylated, switching their fate from proteasomal degradation to release from the cell via unconventional secretion in a CSP-regulated manner [49–52]. Most recently, CSP has also been implicated in a protein degradation pathway that is mechanistically distinct to MAPS: endolysosomal microautophagy [50].

**Figure 1.**
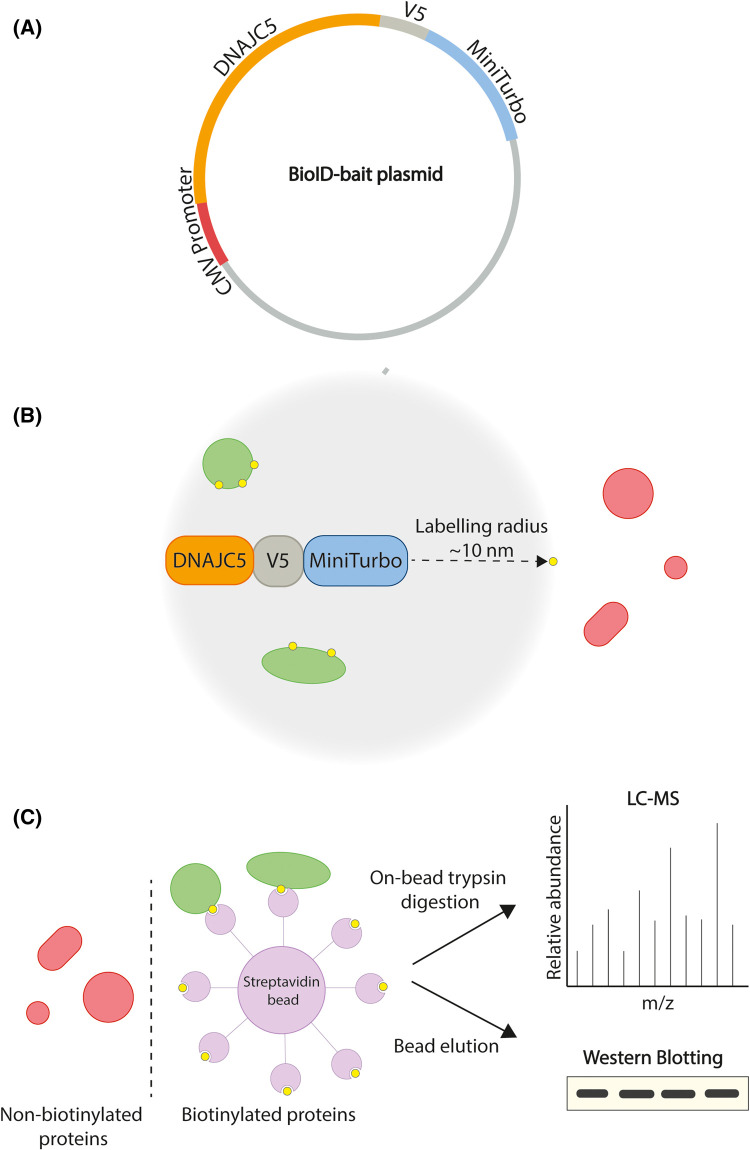
Proximity labelling approach. (**A**) Plasmids are constructed containing cDNA encoding DNAJC5 (wild type or mutant) fused to miniTurbo. (**B**) Following transfection and biotin addition, the miniTurbo enzyme tag labels proteins within 10 nm of the bait with biotin (denoted by yellow circles). (**C**) Cells are lysed and biotinylated proteins isolated using streptavidin beads, and identified by either mass spectrometry or western blotting.

Current evidence suggests that CSP helps to maintain neuronal proteostasis either by directly refolding misfolded client proteins, or by re-routing them for disposal via MAPS and endolysosomal microautophagy. However, little is known about the proteins that CSP interacts with to enable these neuroprotective functions. The relatively small number of CSP-binding proteins identified to date have mostly been discovered via biochemical affinity purification or immunoprecipitation techniques. This is a potential problem, as CSP forms SDS-resistant oligomers after detergent extraction from cellular membranes [17,43,53,54], which would prevent the detection of physiologically relevant protein interactions mediated by binding sites buried within the oligomeric aggregates. The yeast 2-hybrid method is also poorly suited to membrane proteins like CSP, which may explain why screening of brain cDNA libraries using this approach identified only two interacting proteins, both of which are cytoplasmic: Hsc70 [55] and SGT [56]. We, therefore, chose to characterise the CSP interactome using the BioID proximity labelling approach [57] (Figure 1). This enables protein interactions to be detected *in situ* in living cells by fusing the biotin ligase, miniTurbo, onto the protein of interest and identifying biotinylated proteins using liquid chromatography–mass spectrometry (LC–MS). As biotinylation can only occur within a 10-nm radius of the fusion protein [58], equivalent to the size of a typical globular protein, this selectively labels proteins that are in extremely close proximity and hence are likely to be physical binding partners. Here, we report the application of the BioID approach in neuroendocrine PC12 cells to define the protein interactome of wild-type CSP and how this is affected by the L115R ANCL-causing mutation.

## Results

It has previously been established that the addition of fluorescent protein tags on either the N terminus [59,60] or the C terminus [35], of DNAJC5/CSPα does not affect its localisation or chaperone activity. However, the N terminus comprises seven alpha helices, the first of which is a target for phosphorylation that triggers major structural changes; whereas the C terminus is essentially unstructured [61]. Therefore, the miniTurbo tag was fused onto the C terminal domain of CSPα via Gibson assembly (Figure 1), to minimise the possibility of interfering with CSPα structure or function. To investigate how CSP interactions are affected in ANCL, the most common and most extensively studied ANCL-causing mutation, L115R [27,62], was introduced to the miniTurbo-tagged CSP constructs through site-directed mutagenesis. An empty vector containing non-fused miniTurbo was utilised as a negative control, to eliminate proteins which may be randomly biotinylated due to the expression of miniTurbo within the cell, or proteins which have an affinity to the miniTurbo enzyme itself or the neutravidin/streptavidin beads used for the biotin-affinity purification (hereafter referred to as miniTurbo control). An additional negative control was utilised whereby cells expressing the miniTurbo-tagged WT CSP construct were not exposed to exogenous biotin, to eliminate proteins that are pulled down in a biotin-independent, non-specific manner (hereafter referred to as no biotin control).

**Figure 2.**
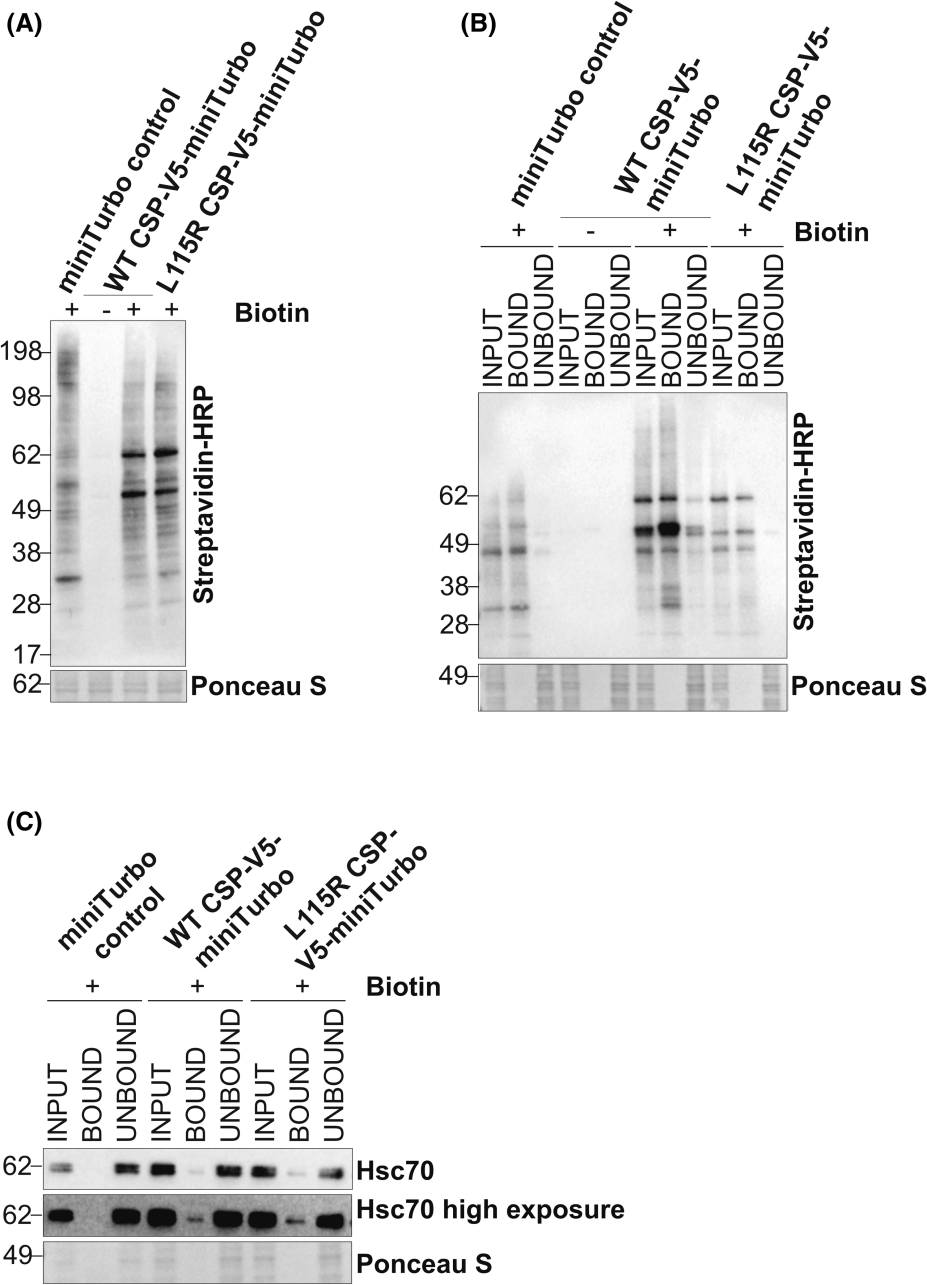
Validation of miniTurbo biotinylation. (**A**) Representative western blot of HEK293T cells transfected with either an empty miniTurbo control vector, or CSP WT/L115R-V5-miniTurbo, either in the presence (+) or absence (−) of biotin. Membranes were probed for streptavidin-HRP, with Ponceau S representing total protein. (**B**,**C**) Representative western blot of lysates from (**A**) following a pull-down of biotinylated protein. Samples loaded include whole cell lysate prior to the pull-down (input), proteins eluted (bound) and the flow-through (unbound). Membranes were probed for streptavidin-HRP (**B**) and Hsc70 (**C**), with Ponceau S representing total protein.

To confirm that the miniTurbo CSP constructs were effectively biotinylating proteins, HEK293T cells transiently transfected with miniTurbo constructs were supplemented with biotin in the cell culture media and analysed by western blotting. Probing with streptavidin-horse radish peroxidase (HRP), which has a high affinity for biotinylated proteins, revealed a strong signal when compared with the no biotin control (Figure 2A). This indicated that the constructs were expressed and capable of biotinylating proteins in a biotin-dependent manner. Next, to validate the efficiency of biotinylated protein capture, the lysates were subjected to biotin-affinity purification, initially utilising Neutravidin beads. Western blotting for streptavidin-HRP revealed an enrichment of biotinylated proteins in the elution (bound), compared with the flow-through (unbound) for the miniTurbo-tagged CSP lysates (Figure 2B). Finally, to ensure that the CSP-miniTurbo constructs were biotinylating genuine CSP interactors, rather than randomly biotinylating proteins within the cell, lysates subjected to biotin-affinity purification were analysed by western blotting utilising antisera against the best-characterised CSP-binding protein, its co-chaperone Hsc70 [55]. As CSP's interaction with Hsc70 has been demonstrated to not be impacted by the ANCL-causing L115R mutation [28,29], it was expected that both WT and L115R CSP-miniTurbo would biotinylate Hsc70. Consistent with this prediction, Hsc70 was eluted from the biotin-affinity purification in both the WT and L115R CSP fused miniTurbo conditions and was absent from miniTurbo control (Figure 2C). This confirmed that the CSP-miniTurbo constructs are not only expressed and enzymically active, but are biotinylating proteins that are known to interact with CSP.

It was noticeable that a large proportion of the biotinylated proteins were not captured by the Neutravidin beads and were, therefore, present in the unbound flow-through, which is particularly visible in the miniTurbo-tagged WT CSP unbound lysates (Figure 2B). To ensure that there was minimal loss of proteins during the BioID process, we focused on optimisation of both the biotinylation of proteins and their subsequent capture/elution by biotin-affinity purification. HEK293T cells transiently transfected with WT CSP-miniTurbo were exposed to biotin concentrations ranging from 0 to 500 μM for 0–24 h. Western blotting the cell lysates with streptavidin-HRP revealed that the levels of biotinylated proteins saturated following exposure to 100 μM biotin (Supplementary Figure S1A). Additionally, exposure to biotin for 24 h yielded more biotinylated proteins compared with the shorter incubation times (Supplementary Figure S1B). In particular, 24 h biotin exposure yielded considerably more biotinylated proteins compared with the 10 min timepoint frequently utilised for miniTurbo BioID protocols [57]. Next, the length of time the proteins were incubated with the biotin-affinity beads was assessed. This revealed that most biotinylated proteins were captured within 1–2 h, after which point it appeared another factor such as bead binding capacity was the limiting factor (Supplementary Figure S2A). Therefore, both the quantity (Supplementary Figure S2B) and type of beads used (Neutravidin versus streptavidin; Supplementary Figure S3) were optimised. It was found that magnetic streptavidin beads eluted an increased quantity of biotinylated protein. Furthermore, unlike the Neutravidin beads, no biotinylated protein was visible in the unbound flow-through when western blotting for streptavidin-HRP when using streptavidin beads (Supplementary Figure S3). Lastly, to ensure there was maximum elution of biotinylated proteins from the beads, elution time was optimised. Through boiling the beads at timepoints from 1 to 30 min, it was observed that most of the biotinylated proteins were eluted after 10 min (Supplementary Figure S4). However, increasing the elution time to 30 min marginally increased the quantity of biotinylated proteins eluted. Overall, our optimised protocol for protein biotinylation involved exposure to 100 μM biotin for 24 h, capture of biotinylated proteins with magnetic streptavidin beads for a minimum of 1 h, with subsequent elution through boiling in SDS for 10 min.

**Figure 3.**
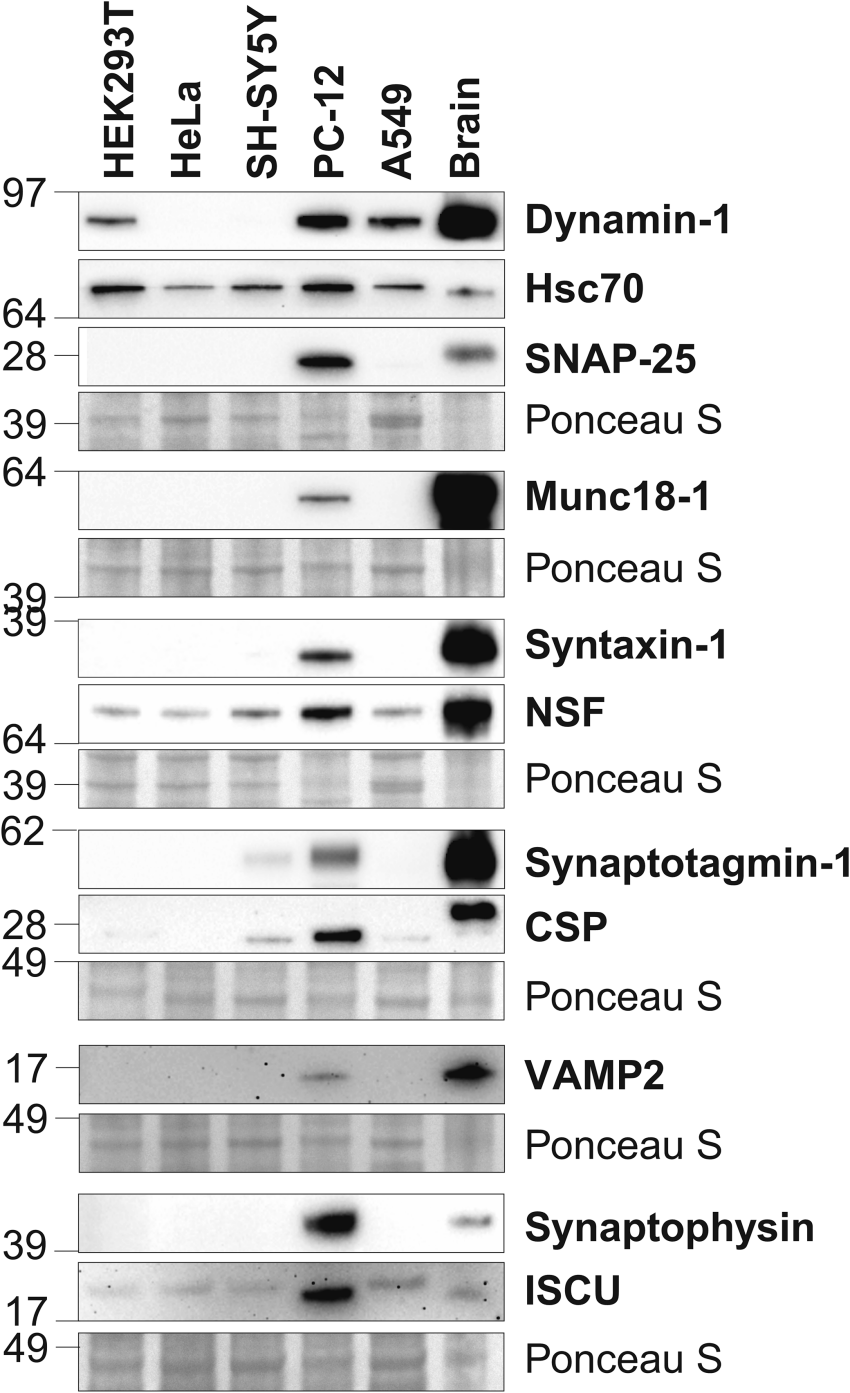
PC12 cells express high levels of CSPα-associated proteins. Western blot of HEK293T, HeLa, SH-SY5Y, PC12 and A549 cell lysates, probing for both reported CSPα-interacting proteins and general synaptic proteins. Rat brain lysates were used as a positive control for antisera binding. Ponceau S staining represents total protein loading.

To determine the most physiologically relevant cell line to use for identifying CSP interactions through LC–MS, various cell lines were analysed by western blotting to assess their expression levels of proteins previously reported to bind to CSP, in addition to general synaptic proteins. Utilising rat brain lysates as a positive control for antisera binding, HEK293T and HeLa cells were compared due to their high transfection efficiencies and ease of culture. Furthermore, HEK293T cells have been used previously to determine CSP interactions utilising BioID [63]. SH-SY5Y neuroblastoma cell lines were included due to their frequent use in the study of neurobiology and neuronal properties [64]. PC12 cells were chosen as they are widely used as a neuronal model of exocytosis, and express relatively high levels of synaptic/secretory vesicle-associated proteins, CSP included [65]. Moreover, PC12 cells have been widely used to study CSP function [17,31,60]. Finally, A549 cells were also compared, given the recent discovery that CSP is required to traffic *Pseudomonas aeruginosa* toxin ExoU from the cytoplasm to the plasma membrane in these cells [8]. Overall, PC12 cells had the highest endogenous expression of all proteins tested (Figure 3), thus it was reasoned that PC12 cells would be the most physiologically relevant cell line to use for identifying neuronal CSP interactions.

**Figure 4.**
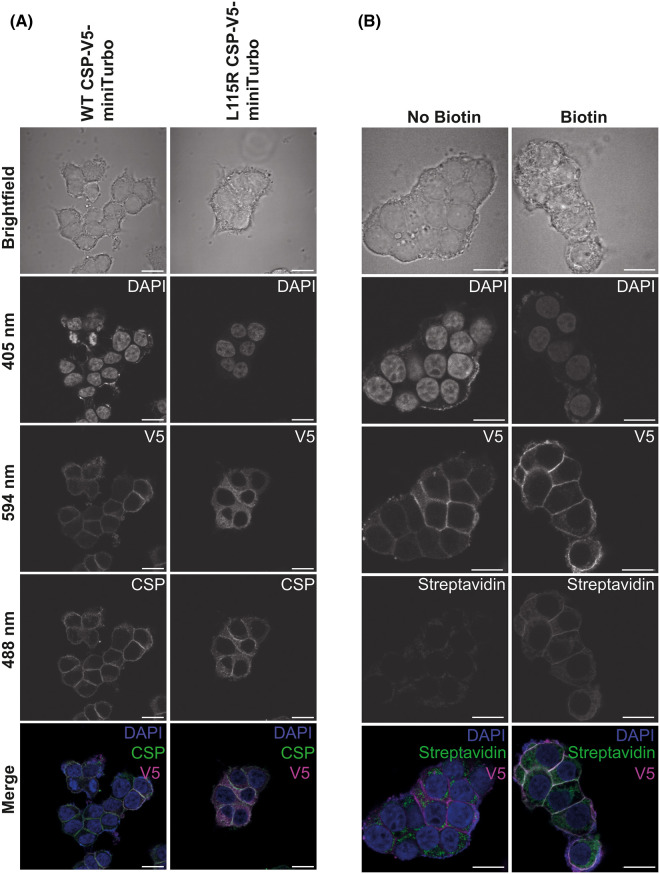
Immunofluorescence of PC12 cells stably transfected with WT/L115R CSP-V5-miniTurbo constructs. Cells were fixed, permeabilised and then probed with DAPI and antisera against the V5 epitope; along with either anti-CSP antibody (**A**) or Alexa488-Streptavidin (**B**) to label biotinylated proteins. Images shown were acquired on a Zeiss Axio Examiner ZI LSM880 confocal microscope, using 405, 488 and 594 nm excitation lasers with 63× objective lens. Scale bars: 10 μm.

We therefore created stable PC12 cell lines expressing WT or L115R CSP fused to miniTurbo, as well as the unfused miniTurbo control construct. Stable cell lines were chosen to provide reproducibility between technical repeats and sufficient transfected cells for proteomic analyses. To investigate whether overexpression of CSP or the miniTurbo tag itself affects CSP localisation, immunofluorescence was carried out. Antisera against the V5 tag was used to reveal localisation of exogenously expressed miniTurbo-tagged CSP, and antisera against CSP was used to identify both endogenous and exogenous CSP (Figure 4A). A high degree of co-localisation was observed between CSP and V5 in cells expressing WT CSP-miniTurbo, primarily near the plasma membrane (which may correspond to docked secretory vesicles) and to a lesser extent throughout the cytoplasm. This suggests that the miniTurbo tag does not interfere with CSP localisation in PC12 cells. The extent of co-localisation between CSP and V5 in the cells stably expressing L115R CSP-tagged miniTurbo was much lower compared with that of the WT. A degree of co-localisation could be observed throughout the cytoplasm. However, the co-localisation with endogenous CSP near the plasma membrane was not observed with V5 labelling. This is consistent with published data showing that ANCL-causing mutations result in CSP mislocalisation [22,26]. Detection of biotinylated proteins in cells expressing miniTurbo-tagged WT CSP using AlexaFluor-streptavidin revealed a change from faint punctate localisation in the no biotin condition to a more widespread distribution that was particularly concentrated near the plasma membrane, where it overlapped with the V5 signal (Figure 4B). Streptavidin fluorescence in cells expressing miniTurbo-tagged WT CSP also co-localised with endogenous CSP near the plasma membrane, in contrast with cells expressing the miniTurbo control (Supplementary Figure S5).

Having established that miniTurbo-tagged WT CSP biotinylates proteins in a biotin-dependent manner and that its cellular localisation is comparable to that of endogenous CSP, the entire complement of proteins biotinylated by miniTurbo-tagged CSP was determined. This involved on-bead tryptic digestion followed by LC–MS and label-free quantification using three independent biological repeats per condition. The resulting data were first analysed by principal component analysis, to assess variance within the datasets (Supplementary Figure S6). This revealed that all three samples for WT CSP miniTurbo were closely clustered and distinct from those of L115R CSP-tagged miniTurbo, miniTurbo and no biotin controls. Proteins identified by LC–MS were then analysed based on their abundance relative to control conditions and statistical significance. Full MS data, including information on quantification, statistics and GO terms are shown in Supplementary Dataset 1. The gene names and UniProt accession numbers of the top 50 proteins enriched in WT or L115R CSP compared with miniTurbo and no biotin controls are listed in Table 1. Functional enrichment analysis of WT and L115R CSP compared with miniTurbo control was performed using DAVID [66] and the results are displayed in Supplementary Dataset 2. Proteins exhibiting reduced abundance in WT or L115R CSP compared with controls were not analysed further, as these represent non-specific and/or endogenously biotinylated proteins.

**Table 1.** Gene names of top 50 LC–MS hits

CSP WT miniTurbo vs.			CSP L115R miniTurbo vs.	
miniTurbo control	No biotin control	CSP L115R miniTurbo	miniTurbo control	CSP WT miniTurbo
Hspa1a* (P0DMW1)	miniTurbo*	C2cd2l* (Q5U2P5)	Hspa1a (P0DMW1)	Acaca* **(P11497)**
Sidt2* (D3ZEH5)	Snap25* (P60881-2)	Snx3* (Q5U211)	Myh11 (E9PTU4)	Mccc1 (F1LP30)
Vamp2* (P63045)	Agfg1* (Q4KLH5)	Arhgdia (Q5XI73)	Sidt2* (D3ZEH5)	Tuba1b (Q6P9V9)
Arhgdia* (Q5XI73)	Csrp1* (P47875)	Stx7* (O70257)	Dnajc5 (A0A0G2JX56)	Nefm (P12839)
Snx3* (Q5U211)	Scamp1* **(P56603)**	Baiap2* (Q6GMN2)	Nudcd2 (Q5M823)	Map1b (P15205)
Stx7* (O70257)	Comt* **(P22734)**	Hn1(Q6AXU6)	Hsph1 (Q66HA8)	Map1a (P34926)
C2cd2l (Q5U2P5)	Hn1* **(Q6AXU6)**	Asap1* **(Q1AAU6)**	Hsp90b1 (Q66HD0)	Pc (P52873)
Stxbp1* (P61765)	Cdv3* **(A0A0G2K0B0)**	Vamp2* (P63045)	Stip1 (O35814)	Prph (P21807)
Pcdh1* (A0A0G2K6T9)	Dpysl2* **(P47942)**	Sept11 (A0A0G2JUL7)	Pcca (A0A0G2K401)	
Nudcd2* (Q5M823)	Txn1* **(P11232)**	Sept8 (G3V9Z6)	Hsp90aa1 (P82995)	
Myh11 (E9PTU4)	Sprr1a* **(G3V755)**	Snap29* (Q9JI56)	Sptan1 (A0A0G2JZ69)	
Sept11 (A0A0G2JUL7)	Ahnak* **(A0A0G2JUA5)**	Ctnnd1* (D3ZZZ9)	Dnajc7 (G3V8B8)	
Scamp1 (P56603)	Sept7* **(D4A0F5)**	Esyt2* (D3ZJ32)	Coro1c (G3V624)	
Dnajc5* (A0A0G2JX56)	Stx7* (O70257)	Stxbp1* (P61765)	Hsp90ab1 (P34058)	
Baiap2* (Q6GMN2)	Hn1l* **(Q5BK20)**	Cdv3* **(A0A0G2K0B0)**	Sptbn1 (G3V6S0)	
Ppid (Q6DGG0)	Snap91* **(F1LRK0)**	Pcdh1 (A0A0G2K6T9)	Aip (Q5FWY5)	
Snap25* (P60881-2)	Picalm* **(Q498N4)**	Basp1* **(Q05175)**	Ppid (Q6DGG0)	
Epb41l2* (D3ZDT1)	Pak2* **(Q64303)**	Eif2a* **(D3ZUV3)**	Atp7a (P70705)	
Esyt2* (D3ZJ32)	Tagln2* **(Q5XFX0)**	Anlnl1 (M0R9N8)	Rps5 (B0BN81)	
Sept9* (F1LN75)	C2cd2l* **(Q5U2P5)**	Ahcyl2 (D3ZWL6)	Acaca (P11497)	
Slc9a3r1* (Q9JJ19)	Esyt1* (Q9Z1X1)	Pebp1 (P31044)	Tuba1b (Q6P9V9)	
Ctnna1* (Q5U302)	Ahcyl2* **(D3ZWL6)**	Ctnna1 (Q5U302)	Trpv2 (A0A0G2JSH6)	
Clk2* (A0A0G2K5Q8)	Nudcd2* (Q5M823)	Slc9a3r1* (Q9JJ19)	Sugt1 (B0BN85)	
Ctnnd1* (D3ZZZ9)	Cttn* **(D3ZGE6)**	Sept9* (F1LN75)	Pdia3 (P11598)	
Add1* (A0A0G2JSM7)	Tpd52l2* **(Q6PCT3)**	Ykt6 (Q5EGY4)	Aco2 (Q9ER34)	
Sept6* (B5DFG5)	Sept11* (A0A0G2JUL7)	Sprr1a (G3V755)		
Sept8* (G3V9Z6)	Pdap1* **(Q62785)**	Snap25* (P60881-2)		
Ykt6 (Q5EGY4)	Arhgdia* (Q5XI73)	Esyt1* **(Q9Z1X1)**		
Anlnl1 (M0R9N8)	Stip1* **(O35814)**	Sept7 (D4A0F5)		
Basp1* (Q05175)	Snx3* (Q5U211)	Sept6* (B5DFG5)		
Pebp1 (P31044)	Cct8* (D4ACB8)	Scamp1 (P56603)		
Trpv2* (A0A0G2JSH6)	Nudc* (Q63525)	Pdap1* **(Q62785)**		
Gnas* (Q63803)	Vamp2* (P63045)	Chmp2b* **(F1M8B7)**		
Snap29 (Q9JI56)	Sytl4* **(G3V6G6)**	Rab11b (O35509)		
Rab11b* (O35509)	Clint1* **(Q6DGF2)**	Arhgap1 (D4A6C5)		
Yes1* (F1LM93)	Crip2* (P36201)	Sh3gl1* **(O35964)**		
Sept7 (D4A0F5)	Epb41l3* **(A3E0T0)**	Necap2* **(Q6P756)**		
Atp7a (P70705)	Mllt4* **(O35889)**	Camlg* **(Q6DGG9)**		
Pkn2* (F1LPA4)	Ubap2l* **(E9PTR4)**	Chmp4b* (M0RCH6)		
Atp2b4* (Q64542-4)	Pdlim1* **(P52944)**	Sept2 (Q91Y81)		
Sptbn1* (G3V6S0)	Stxbp1* (P61765)	Pdcd5* **(D4ADF5)**		
H2ac (P0CC09)	Madd* **(O08873)**	Epb41l2* **(D3ZDT1)**		
Vti1b* (P58200)	Eif4b* **(Q5RKG9)**	Crip1 (P63255)		
Arpp19 (Q712U5)	Esyt2* (D3ZJ32)	**Add1 (A0A0G2JSM7)**		
Fam129b* (B4F7E8)	Arhgap1* (D4A6C5)	Picalm* **(Q498N4)**		
Hsph1 (Q66HA8)	Dbnl* **(Q9JHL4-2)**	Add3 (D3ZCH7)		
Dnajb1* (B0K030)	Snap29* (Q9JI56)	Rab11fip1 (Q3B7T9)		
Rab3d (Q63942)	Sncb* (Q63754)	Ist1* **(Q568Z6)**		
Tub* (O88808)	Sept9* (F1LN75)	Fcho2* **(D3ZYR1)**		
miniTurbo*	Eps15l1* **(D3ZJR1)**	Ccdc124* **(D3ZUL1)**		

Proteins with >1.5-fold increase in abundance are listed by gene name and UniProt accession number in order of greatest fold-change. Statistically significant hits are highlighted in bold and marked by an asterisk.

**Figure 5.**
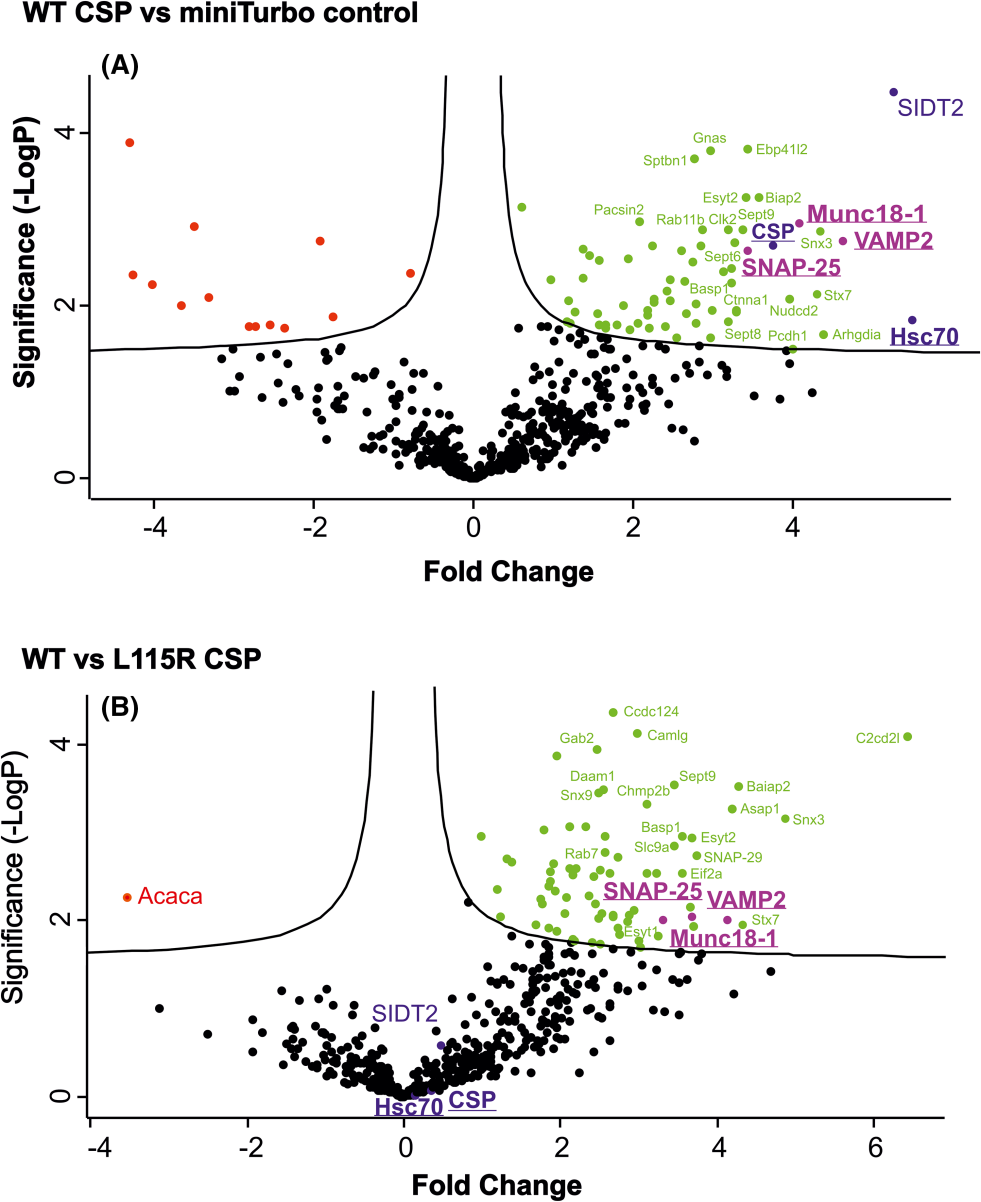
Volcano plot of LC–MS hits from biotin-affinity purification. Comparison of proteins enriched in PC12 cells expressing WT CSP-miniTurbo versus miniTurbo control (**A**) and L115R CSP-miniTurbo (**B**). Proteins identified in the proteomic analysis are distributed according to fold abundance change and statistical significance. The curved line indicates a false discovery rate of 0.05. Proteins enriched in WT CSP are shown in green, with the exception of selected hits of interest, which are colour co-ordinated based on whether they were affected (VAMP-2, SNAP-25, Munc18-1, in magenta) or unaffected (Hsc70, CSP, SIDT2, in blue) by the L115R mutation. Proteins enriched in the miniTurbo control and L115R mutant are shown in red.

**Figure 6.**
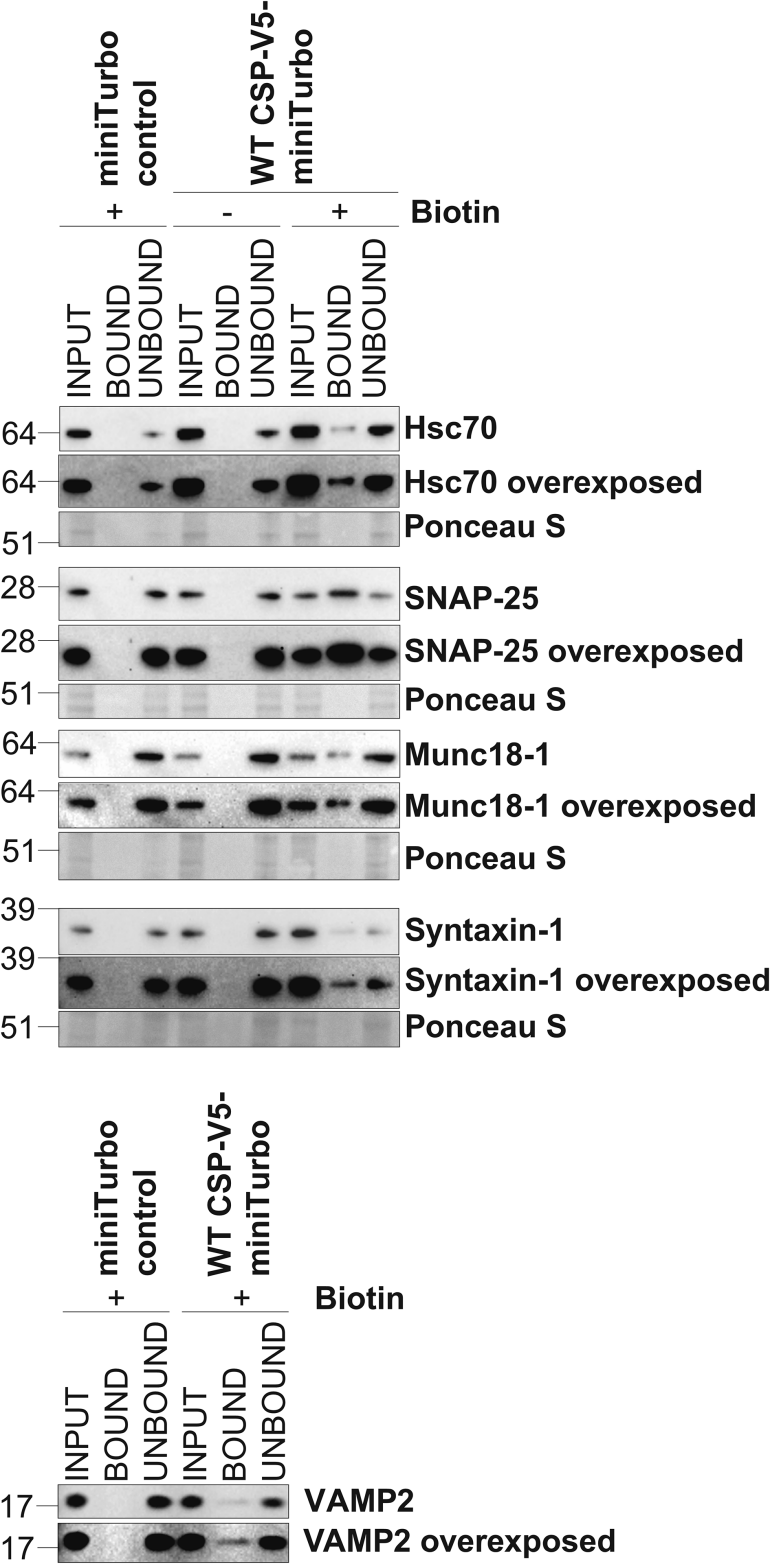
Validation of WT CSP LC–MS hits through western blotting. PC12 cells stably transfected with miniTurbo control or WT CSP-V5-miniTurbo, with a biotin-free control, were subjected to pull-down of biotinylated proteins. Samples loaded include whole cell lysate prior to the pull-down (input), protein bound to the streptavidin beads (bound) and the flow-through (unbound). Membranes were probed for various hits of interest from the LC–MS results, with total protein loaded represented by Ponceau S staining.

**Figure 7.**
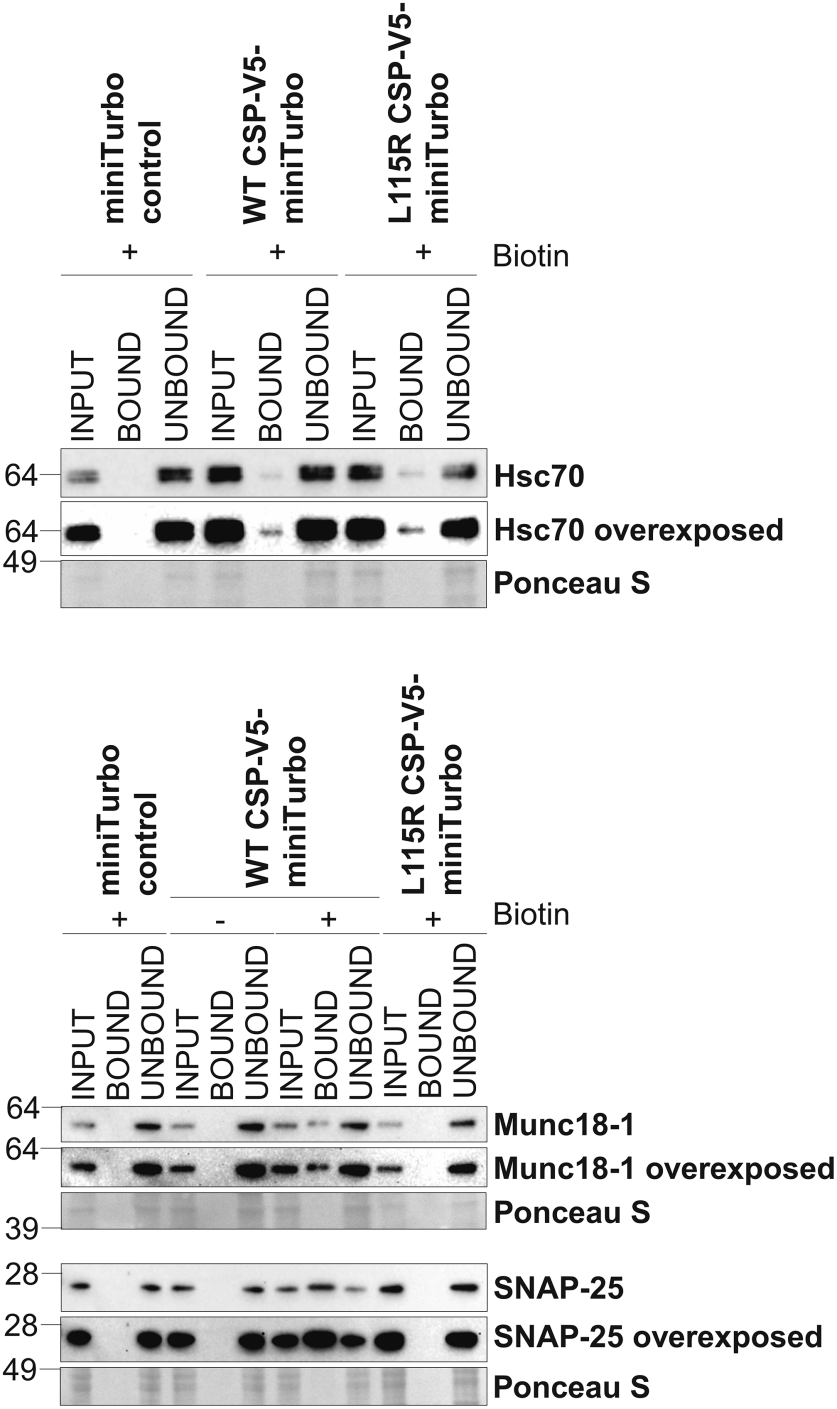
Validation of WT/L115R CSP LC–MS hits through western blotting. PC12 cells stably transfected with miniTurbo control or WT/L115R CSP-V5-miniTurbo and subject to pull-down of biotinylated protein. Samples loaded include whole cell lysate prior to the pull-down (input), protein bound to the streptavidin beads (bound) and the flow-through (unbound). Membranes were probed for Munc18-1, SNAP-25 and Hsc70, with total protein loaded represented by Ponceau S staining.

The single most abundant protein when comparing miniTurbo-tagged WT CSP with the miniTurbo control was CSP's co-chaperone Hsc70 (gene name *hspa1a*), which was 5.5-fold enriched (Figure 5A). Several other previously identified CSP interactors were also identified, including SNAP-25 and VAMP2. Interestingly, many biotinylated proteins were detected that have not been reported as CSP-binding proteins. Notably, the essential exocytosis protein Munc18-1 (gene name *stxbp1*) was increased in abundance 4.1-fold in the miniTurbo-tagged WT CSP condition, compared with the miniTurbo control. A subset of these potential interactions identified through LC–MS were further validated through western blotting, including Hsc70, SNAP-25, VAMP2 and Munc18-1 (Figure 6). Whilst the lack of Hsc70 observed in the miniTurbo control in Figures 6 and 7 could conceivably be due to a lower level of input protein, the Hsc70–CSP interaction has been experimentally demonstrated by various groups over many years [15,29,32,33,42,55], and hence is likely to be authentic. In addition, the well-established interaction between CSP and syntaxin-1 [6,15,38,39] was confirmed by western blotting, although it did not reach the threshold for detection in our LC–MS analysis.

**Figure 8.**
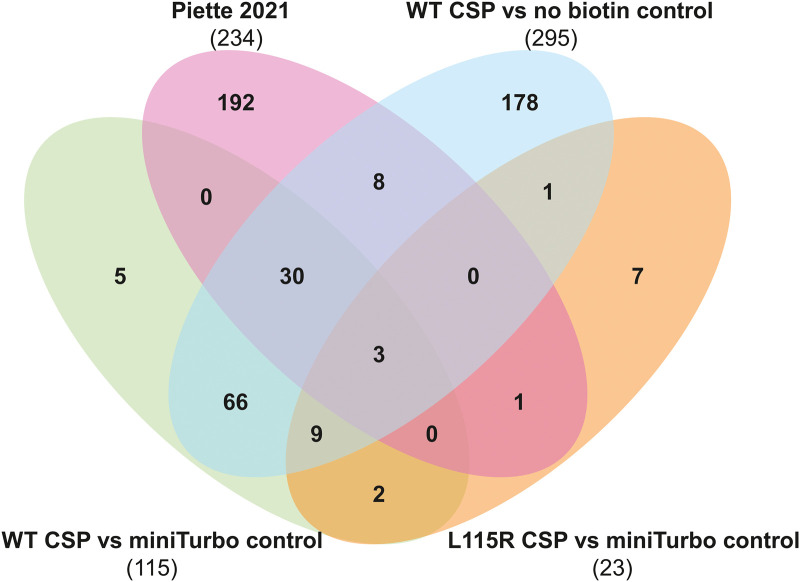
Venn diagram of BioID hits common to this study and other published data. Overlap was assessed between proteins increased in abundance by >1.5-fold in WT CSP compared with the miniTurbo control and no biotin control, in L115R CSP compared with the miniTurbo control, and LC–MS results on WT CSP from Piette et al. [63].

When comparing the BioID hits between miniTurbo-tagged WT and L115R CSP, several synaptic exocytosis proteins were significantly reduced in abundance in the L115R mutant, including SNAP-25, Munc18-1 and VAMP2 (Figure 5B). Western blot validation confirmed that the biotinylation of Munc18-1 and SNAP-25 in the WT CSP condition was reduced in the L115R mutant (Figure 7). This could not be simply due to reduced expression of the L115R CSP miniTurbo construct, as LC–MS analysis revealed no change in Hsc70 levels between the WT and L115R mutant CSP conditions, which was subsequently validated by western blotting (Figure 7). This suggests that the ANCL L115R mutation does indeed affect interactions between CSP and these binding clients. There was only one protein significantly enriched in the L115R CSP mutant, compared with WT CSP: acetyl-CoA carboxylase alpha (gene name *acaca*; Figure 5B). However, this naturally biotinylated enzyme (also known as biotin carboxylase) is typically excluded from LC–MS results in BioID studies [67], and was also present in the miniTurbo control condition. Hence, acetyl-CoA carboxylase alpha is likely an artefact that binds to streptavidin beads via its endogenous biotin moiety, rather than via miniTurbo-driven biotinylation. Finally, in order to assess how our LC–MS results correspond to published data, the results were correlated with that of published data investigating WT CSP interactions using BioID in HEK293T cells [63]. This revealed that 33 of the 108 proteins we found to be enriched in the miniTurbo-tagged WT CSP condition compared with both miniTurbo and no biotin controls were also identified by Piette et al. [63] (Figure 8). Details of the proteins present across datasets from the Venn diagram in Figure 8 are listed in Supplementary Table S3.

## Discussion

In this study, we used BioID to define the CSP interactome in a neuronal model cell line, in order to shed light on potential substrate proteins that may underlie its evolutionarily conserved neuroprotective role(s). BioID was chosen as it labels proteins *in situ* in their physiological subcellular compartments within living cells. This avoids potential artefacts that can arise with conventional pull-downs and co-immunoprecipitation approaches, which particularly affects membrane-localised proteins that aggregate after detergent solubilisation such as CSP. One caveat of BioID is that it does not directly demonstrate that biotinylated proteins bind to the bait protein, only that they are in close proximity (<10 nm). However, the most enriched biotinylated protein in WT CSP compared with miniTurbo control was Hsc70, thereby confirming the well-established direct interaction between CSP and its co-chaperone [55]. CSP's best-characterised client for refolding, SNAP-25 [35,42,68] was also verified through our unbiased proximity labelling approach; in addition to the reported CSP interacting protein, VAMP2 [37]. This provides reassurance that BioID proteomic screening can identify genuine CSP interactions. Not all previously published CSP-binding proteins were detected, however. There are several potential explanations for this. Our stringent MS identification criteria, requiring multiple peptides to be detected in every biological repeat, may have filtered out some genuine hits — for example, syntaxin-1, which was validated here by western blotting. In addition, interactions that are dependent on post-translational modifications would be missed if the recombinant bait protein is not sufficiently modified — for example, binding of 14-3-3 requires CSP phosphorylation on serine-10 [45]. We may also have failed to detect some bona fide interacting proteins because they lack surface-exposed lysine residues that are accessible to the miniTurbo ligase, which is an inherent limitation of the BioID approach. Alternatively, it is possible that some previously reported interactions are a consequence of the non-physiological, aggregation-prone conformation of CSP when removed from membranes, and so may not reflect *in vivo* binding proteins.

In addition to confirming previously reported interactions, our LC–MS analysis revealed over 100 new potential CSP-binding proteins. The vast majority of these are cytoplasmic proteins or membrane proteins with domains that project into the cytosol, consistent with CSP's known topology as a membrane-associated cytoplasmic protein. However, a few hits are likely to be false positives — for example, H2ac (Table 1) encodes histone H2A, a nuclear protein that is therefore unlikely to be a bona fide CSP-interacting protein. Around a third of our hits were also identified in a recent BioID screen of all DnaJ-domain-containing proteins in HEK293 cells [63]. However, as shown in Figure 2, HEK293 cells express low levels of many of CSP's previously reported prey proteins, relative to PC12 cells used in this study. This, as well as technical differences in the BioID methodologies used, could account for the relatively small overlap in results. Nevertheless, if homologous proteins were considered to match, there would be better overlap between the datasets. For example, Piette et al. identified SNAP-23 and VAMP8 in their LC–MS analysis, which are homologues of the neuronal SNAREs SNAP-25 and VAMP2 respectively, identified in this study. Furthermore, both studies identified the non-exocytotic SNAREs syntaxin-7 and the VAMP homologue ykt6, suggesting that CSP interacts with SNAREs in various subcellular compartments. The identification of so many SNAREs by us (VAMP2, ykt6, vti1b, SNAP-25, SNAP-29, syntaxin-1, syntaxin-7) and by Piette et al. (SNAP-23, VAMP8, ykt6, syntaxin-7) is striking given that mammalian genomes only encode 36 SNARE proteins in total [69]. Indeed, unbiased functional annotation clustering analysis of our WT CSP BioID hits using DAVID revealed that the most significantly enriched GO terms for molecular function, cellular component and biological process were: SNARE activity, SNARE complex and vesicle fusion, respectively (Supplementary Dataset 2). Intracellular membrane fusion events throughout the secretory and endocytic pathways are driven by SNAREs, but also require the function of the equally conserved Rab and Sec1/Munc18 (SM) protein families [69]. Various Rab proteins were identified in our BioID study (Rab3D, Rab7A, Rab11B, Rab23) and Rab 7A was also identified by Piette et al. [63]. A further link between CSP and the universal membrane fusion machinery comes from our identification by BioID screening and western blot validation of Munc18-1, a neuronal SNARE-binding SM protein that is essential for synaptic vesicle exocytosis [70]. Taken together, these BioID hits suggest that a major cellular function of CSP is to modulate intracellular membrane fusion events. In addition, functional clustering analysis revealed various other significantly enriched GO terms, including α-catenin binding, endocytic vesicle, clathrin binding and clathrin coat assembly (Supplementary Dataset 2). This may be relevant to published work showing that CSPα partially localises to endolysosomal structures [7] and plays a functional role in endocytic recycling [42,71].

Analysis of BioID hits from PC12 cells expressing CSP L115R-miniTurbo revealed that several synaptic proteins were significantly reduced in abundance compared with WT CSP. In contrast, the levels of various other proteins remained unchanged between the WT and L115R mutant, notably Hsc70 — an interaction that has previously been shown to persist in ANCL mutant CSP [28,29]. This suggests that the reduction seen by both LC–MS and western blotting of SNAP-25, Munc18-1 and VAMP2 is likely to be a direct result of the L115R mutation itself, rather than a reduction in CSP L115R-miniTurbo transgene expression. Indeed, a major advantage of the label-free quantification approach used in our proteomic analysis is that it inherently takes account of variability in miniTurbo expression levels (and hence biotinylation levels) between different constructs and biological replicates [72]. Furthermore, the reduction in SNAP-25 biotinylation in the L115R mutant is consistent with studies utilising cultured neurons, which demonstrated through co-immunoprecipitation that L115R/L116Δ CSP interaction with SNAP-25 is severely reduced compared with WT CSP [29]. This is consistent with the mislocalisation of L115R CSP observed here and previously reported in the literature [22,23]. Contrary to findings in cultured neurons whereby L115R CSP (but not WT CSP) was identified to interact with ISCU [29], LC–MS did not identify ISCU in the complement of proteins biotinylated by miniTurbo-tagged L115R CSP. However, this should not be interpreted as evidence against the proposed interaction between ISCU and ANCL mutant CSP, as there are various reasons why bona fide interactions may be missed in BioID screening (explained above). Although relatively few proteins were enriched >1.5-fold in CSP L115-miniTurbo compared with miniTurbo control, these may represent potential CSP interacting proteins involved in endolysosomal microautophagy, as the ANCL-causing L115R mutation has no effect on this protein degradation pathway [50]. One such protein, SIDT2, is a lysosomal membrane protein that has been shown to protect against mRNA-encoding mutant Huntingtin [73]. Intriguingly, CSP has previously been shown to bind to mutant (but not WT) Huntingtin protein [74]. Further studies are required to investigate if the putative CSP–SIDT2 interaction is relevant to CSP's L115R-resistant role in endolysosomal microautophagy and its ability to ameliorate neurodegeneration.

Although the proximity labelling analysis reported here has shed light on potential CSP–protein interactions that may be affected in ANCL, there are many limitations of our study. For example, only a few of these interactions could be validated through western blotting, due to limitations in antisera availability and/or specificity, and so may not all be authentic CSP-binding proteins. In addition, our proteomics approach involved stable overexpression of transgenic WT and L115R CSPα in PC12 cells, which already contain high levels of endogenous WT CSPα. Hence, these cells may be partially protected from mutation-associated phenotypes by the endogenous WT protein. Significant work would be required to address this, by first creating CSP knockout PC12 cells and then rescuing these with WT or mutant CSPα (preferably at single-copy level, to mirror the human condition). It will also be important in the future to investigate whether the putative novel interactions reported here are functionally significant, as has been done previously for the SNAP-25 interaction using CSP knockout mice [35,36]. Thus, considerable future work is needed to independently validate the potential novel interactions identified here, to determine if they bind directly to CSP, and to investigate their functional relevance.

## Materials and methods

All reagents were purchased from Sigma unless stated otherwise.

### Cell culture

Cells were cultured at 37°C in 5% CO_2_ in the following media.

#### HEK293T and HeLa

High glucose Dulbecco's modified Eagle's medium (DMEM), supplemented with 10% (v/v) foetal bovine serum, 1% (v/v) penicillin/streptomycin and 1% (v/v) non-essential amino acids.

#### PC12

High glucose DMEM, or RPMI 1640 media, supplemented with 10% (v/v) horse serum, 5% (v/v) foetal bovine serum, 1% (v/v) penicillin/streptomycin and 1% (v/v) non-essential amino acids. For stably transfected PC12 cell lines, culture media was additionally supplemented with 500 μg/ml G418 to maintain selection for the miniTurbo constructs.

#### SH-SY5Y

DMEM-F12 supplemented with 10% (v/v) foetal bovine serum, 1% (v/v) penicillin/streptomycin and 1% (v/v) non-essential amino acids.

#### A549

Ham's F-12K supplemented with 10% (v/v) foetal bovine serum and 1% (v/v) penicillin/streptomycin.

### Cloning of miniTurbo plasmids

Initial biotinylation plasmid cloning was based on a previously described [30,31] pcDNA DEST40 untagged wild-type human CSPα plasmid, kindly gifted by Professor Luke Chamberlain. A *XhoI* restriction site was inserted immediately upstream of the stop codon for CSPα, in order to create a fusion protein, using a Q5® Site-Directed Mutagenesis Kit (New England Biolabs). The L115R mutation was introduced through site-directed mutagenesis, using a QuikChange II site-directed mutagenesis kit (Agilent Technologies). Gibson assembly using the NEBuilder kit (New England Biolabs) was employed to introduce the V5 tag and the miniTurbo protein immediately downstream of both wild-type and mutant CSP plasmids, utilising V5-hBirA(64-321) ‘miniTurbo’ plasmid (Addgene plasmid #107170) [57]. All primers used in Gibson assembly and mutagenesis were synthesised by Sigma-Genosys and are described in Supplementary Table S1.

### Transfections

Cells were seeded in six-well plates 24 h prior to transfection, to 70% confluency at the time of transfection. miniTurbo plasmid constructs were transfected with Lipofectamine® 2000 reagent diluted 1:30 in OptiMEM® medium, added to 2 μg plasmid DNA diluted in OptiMEM and incubated for 30 min at room temperature. The DNA-lipid complex was added to cells following aspiration of media, and incubated at 37°C in 5% CO_2_, following which time, fully supplemented DMEM was added. For generation of stable cell lines, 24 h post transient transfection cell culture media was replaced with fully supplemented media containing 500 μg/ml G418, which was replaced every 2 days until 100% of untransfected control cells had died. After which point, 500 μg/ml G418 was supplemented in the culture medium to maintain selection for the miniTurbo constructs.

### miniTurbo biotinylation

Biotin was dissolved in serum-free DMEM to create a 20× stock solution. This was then added to serum-containing cell media at 1 in 20 dilutions, resulting in a final concentration of 100 μM exogenous biotin, unless stated otherwise. Biotin supplementation was added overnight, either 4 h post-transfection for transient transfections, or 24 h after seeding for stable cell lines, unless stated otherwise. Biotin-free and empty miniTurbo constructs with biotin supplementation were used as negative controls.

### Biotinylated protein immunoprecipitation

This was based on previously published protocols for BioID [57,75], with modifications. Cells were lysed in RIPA buffer (50 mM Tris pH 8, 150 mM NaCl, 5 mM EDTA, 0.5% sodium deoxycholate, 0.1% SDS, 1% Triton X-100) with protease inhibitor cocktail containing EDTA (Roche) diluted 1:100, and clarified at 4°C for 10 min at 16 000 ***g***. Prior to the addition of cell lysates, the NeutrAvidin/StreptAvidin beads were washed twice for 10 min in RIPA buffer. Fifty microlitres of normalised lysates were used as the input fraction, and the remaining lysates were then added to the washed beads at a ratio of 10 μl of NeutrAvidin/StreptAvidin beads per 100 μg of protein and incubated at 4°C on a rotator overnight, unless stated otherwise. The following day the supernatant was collected as the unbound fraction. The beads were then washed twice in RIPA buffer, once in 1 M KCl, once in 0.1 M Na_2_CO_3_, once in 2 M urea in 10 mM Tris HCl pH 8, then again twice in RIPA buffer. The beads were then eluted in 100 μl 2× Laemlli buffer (65.8 mM Tris–HCl, pH 6.8, 2.1% SDS, 26.3% (w/v) glycerol, 0.01% bromophenol blue) at 95°C for 10 min, and the supernatant collected as the bound fraction.

### SDS–PAGE and western blotting

Lysates for western blotting were diluted 1:1 in Laemlli buffer, vortexed and heated to 95°C for 10 min, prior to separation by SDS–PAGE and transfer onto nitrocellulose membranes. The transferred proteins were then rapidly stained for total protein with Ponceau S solution before immunoblotting. Details of the antisera used are shown in Supplementary Table S2. Nitrocellulose membranes re-probed with different primary antibodies were stripped by incubation in stripping buffer (20 mM glycine, 140 mM NaCl, pH 2.5) for 30 min. Western blots were visualised through incubation with HRP-labelled secondary antibodies at 1/1000 dilution and subsequent imaging with Clarity™ Western ECL Blotting Substrates (Bio-Rad) and imaged using a Bio-Rad ChemiDoc XRS using ImageLab software. Relative protein quantification was performed using ImageJ software.

### Immunofluorescence

PC12 cells grown on poly-l-lysine-coated coverslips were fixed in 4% paraformaldehyde, washed in phosphate-buffered saline (PBS) and permeabilised in PBT (PBS containing 3% bovine serum albumin and 0.1% Triton X-100). Primary antibodies were incubated for 1 h in PBT at 1/200 dilution; secondary antibodies conjugated to Alexa594 and Alexa488-Streptavidin were incubated for 1 h in PBT at 1/400 dilution. Coverslips were air dried and then mounted in ProLong Gold mounting medium containing DAPI (Thermo Fisher) to visualise DNA. Imaging was performed on a Zeiss Axio Examiner ZI LSM880 confocal microscope, using 405, 488 and 594 nm excitation lasers with 63× objective lens. Image analysis was performed using Zen software (Zeiss).

### Mass spectrometry

#### Sample preparation

To prepare samples for analysis by mass spectrometry, 80% of the streptavidin bead slurry from the final stages of immunoprecipitation, instead of boiling in SDS, was washed twice in 50 mM Tris HCl buffer, pH 7.5. This was then followed by two further washes in 2 M urea/50 mM Tris, pH 7.5 and then incubation in 2 M urea/50 mM Tris, pH 7.5 with 1 mM dithiothreitol (DTT) and 0.4 μg trypsin at 25°C for 1 h, with shaking. The subsequent supernatant was collected and the beads were further washed twice in 2 M urea/50 mM Tris, pH 7.5 and the washes were combined with the supernatant collected from the on-bead digest and reduced with 4 mM DTT at 25°C for 30 min, with shaking. The sample was then alkylated with 10 mM iodoacetamide in the dark at 25°C for 45 min, with shaking. A further 0.5 μg of trypsin was then added to complete the digestion at 25°C overnight, with shaking. Lastly, the sample was acidified with formic acid so that the sample contains ∼1% formic acid, then evaporated to dryness overnight using a ThermoFisher Savant Speedvac SC210A at ambient temperature.

#### NanoLC–ESI–MS/MS analysis

Reversed-phase chromatography was used to separate tryptic peptides prior to mass spectrometric analysis. Two columns were utilised, an Acclaim PepMap μ-precolumn cartridge 300 μm i.d. × 5 mm 5 μm 100 Å and an Acclaim PepMap RSLC 75 μm × 50 cm 2 μm 100 Å (Thermo Scientific). The columns were installed on an Ultimate 3000 RSLCnano system (Thermo Scientific). Mobile phase buffer A was composed of 0.1% formic acid in water and mobile phase B of 0.1% formic acid in acetonitrile. Samples were loaded onto the μ-precolumn equilibrated in 2% aqueous acetonitrile containing 0.1% trifluoroacetic acid for 5 min at 10 μl min^−1^ after which peptides were eluted onto the analytical column at 250 nl min^−1^ by increasing the mobile phase B concentration from 8% B to 25% over 36 min, then to 35% B over 10 min, and to 90% B over 3 min, followed by a 10 min re-equilibration at 8% B.

Eluting peptides were converted to gas-phase ions by means of electrospray ionisation and analysed on a Thermo Orbitrap Fusion (Q-OT-qIT, Thermo Scientific). Survey scans of peptide precursors from 375 to 1575 *m*/*z* were performed at 120 K resolution (at 200 *m*/*z*) with a 50% normalised AGC target and the max injection time was 150 ms. Tandem MS was performed by isolation at 1.2 Th using the quadrupole, HCD fragmentation with normalised collision energy of 33, and rapid scan MS analysis in the ion trap. The MS2 was set to 50% normalised AGC target and the max injection time was 200 ms. Precursors with charge states 2–6 were selected and sampled for MS2. The dynamic exclusion duration was set to 45 s with a 10 ppm tolerance around the selected precursor and its isotopes. Monoisotopic precursor selection was turned on. The instrument was run in top speed mode with 2 s cycles.

#### Data analysis

The raw data were searched using MaxQuant (version 2.0.3.0) against the *Rattus norvegicus* database (www.uniprot.org/proteomes) and the MaxQuant common contaminant database. In addition, sequences for the non-native miniTurbo and CSP L115R proteins were manually added to the database. For database searches, enzyme specificity was set to trypsin with up to two missed-cleavages and carbamidomethylation of cysteines was set as a fixed modification. Oxidation of methionine and acetylation of the protein N terminus were added as variable modifications. Protein abundance was estimated using the label-free intensity quantification algorithm in MaxQuant [72]. To analyse the results, Perseus software (version 2.03.0) was used. Three independent biological replicate samples for each condition were used for the analysis. Functional enrichment analysis of identified proteins enriched >1.5 fold in WT and L115R CSP compared with miniTurbo control was performed using DAVID [66].

## Data Availability

The mass spectrometry proteomics data have been deposited to the ProteomeXchange Consortium via the PRIDE [76] partner repository with the dataset identifier PXD037258 and 10.6019/PXD037258.

## Open Access

Open access for this article was enabled by the participation of the University of Liverpool in an all-inclusive *Read & Publish* agreement with Portland Press and the Biochemical Society under a transformative agreement with JISC.

## Abbreviations

ANCL: adult-onset neuronal ceroid lipofuscinosis
CSP: cysteine string protein
DMEM: Dulbecco's modified Eagle's medium
DTT: dithiothreitol
HRP: horse radish peroxidase
LC–MS: liquid chromatography–mass spectrometry
MAPS: misfolding-associated protein secretion
PBS: phosphate-buffered saline
