# Supplementary material for: Proximity labelling reveals effects of disease-causing mutation on the DNAJC5/cysteine string protein α interactome
Source: Biochem J. 2024 Jan 29;481(3):141–60. doi: 10.1042/BCJ20230319 (PMC10903463; doi:10.1042/BCJ20230319)
Supplement: Supplementary Material 1 [file BCJ-481-141-s1.pdf]

Supplementary Table S1. List of Oligonucleotides used for Cloning.

Oligonucleotide overhangs for ligation are indicated in lowercase letters.

Oligo name	Forward (F) / Reverse (R)	Sequence 5' to 3'
**V5-MiniTurbo**	F	accccagctaccacactgacgggttcaaccTCGAGCGCCACCATGGACTACAAG
	R	agggataggcttaccttcgaaccgcgggccCCTTGAGCTCGAGATCTGAGTCC
**XhoI mutagenesis**	F	CTCGAGTAACCCAGCTTTCTTGTAC
	R	GTTGAACCCGTCAGTGTG
**L115R mutagenesis**	F	TTTGTCTTCTGCGGCCGTCTCACGTGCTGCTAC
	R	GTAGCAGCACGTGAGACGGCCGCAGAAGACAAA
	R	ATGGTGGCGGCCGCATAGTTGAACCCGTCAGTGTGGT

Supplementary Table S2. Primary antibodies used in western blotting.

**Target Protein**	**Working dilution**	**Antibody species**	**Manufacturer**	**Reference or Catalogue #**
DNAJC5/CSPα	1:1000	Sheep	Morgan lab	Evans *et al* (2006) *J. Biol. Chem.* **281**, 1564
Dynamin-1	1:1000	Mouse	BD Biosciences	610245
Hsc70	1:5000	Mouse	Sigma	H5147
ISCU	1:5000	Rabbit	Novus Biologicals	NBP2-14998
Munc-18-1	1:1000	Mouse	BD Biosciences	610337
NSF	1:1000	Mouse	Tagaya lab	Tagaya *et al* (1993) *J. Biol. Chem.* **268**, 2662
Streptavidin-HRP	1:1000	N/A	Sigma	RPN1231
SNAP-25	1:10000	Mouse	Synaptic systems	111 111
Synaptophysin	1:1000	Mouse	Sigma	S5768
Synaptotagmin-1	1:1000	Mouse	Synaptic systems	105 011
Syntaxin-1	1:10,000	Mouse	Abcam	ab3265
VAMP2	1:2000	Rabbit	Takahashi lab	El Far *et al* (1995) *FEBS Lett.* **361**, 101
V5 tag	1:1000	Mouse	Thermo-Fisher	R960-25

Supplementary Table 3 BioID hits from this study and published data. Comparison of LC-MS hits with >1.5-fold upregulation across conditions compared within study and/or with published data from Piette et al 2021. Details of datasets compared are indicated in bold at the top of each column.

**Piette et al., 2021**	**Piette et al., 2021**	**WT CSP vs miniTurbo control**	**CSP WT vs miniTurbo Control**	**WT CSP vs miniTurbo control**
**CSP WT vs miniTurbo Control**	**CSP WT vs miniTurbo Control**	**WT CSP vs no biotin control**	**CSP WT vs no biotin Control**	**L115R CSP vs miniTurbo control**
**L115R CSP vs miniTurbo Control**	**CSP WT vs no biotin Control**	**L115R CSP vs miniTurbo Control**		
atp7a	add1	coro1c	alkbh3	myh11
sptbn1	add3	dnajc5	anlnl1	pdia3
stip1	ap3d1	hspa1a	arfgap3	
	arhgap1	hsph1	arhgdia	
	atp2b4	nudcd2	arpp19	
	baiap2	ppid	asap1	
	basp1	sidt2	c2cd2l	
	cdc42bpb	sugt1	camlg	
	ctnna1	trpv2	cc2d1a	
	ctnnd1		ccdc124	
	daam1		cdc37	
	dlg1		chmp2b	
	epb41l2		chmp4b	
	esyt2		clk2	
	itsn1		crip1	
	jup		dmxl2	
	map4k5		dnajb1	
	mark2		ehd3	
	pacsin2		ehd4	
	pak4		epb41l3	
	pard3		epn2	
	pkn2		esyt1	
	rab11fip1		fabp5	
	rab7a		fam129b	
	scamp1		fcho2	
	stub1		gnas	
	stx7		h2ac	
	tpd52l2		hspa4	
	yes1		lasp1	
	ykt6		miniTurbo	
			palm	
			pcdh1	
			pdcd5	
			pebp1	
			picalm	
			pkp3	
			plekha2	
			ppme1	
			rab11b	
			rab23	
			rab3d	
			rgd1559896	
			sept11	
			sept2	
			sept6	
			sept7	
			sept8	
			sept9	
			sh3gl1	
			slc9a3r1	
			snap25	
			snap29	
			snap91	
			snx1	
			snx3	
			snx9	
			sprr1a	
			stxbp1	
			tagln2	
			tppp	
			tub	
			ube2m	
			vamp2	
			vat1	
			vti1b	
			zwint	

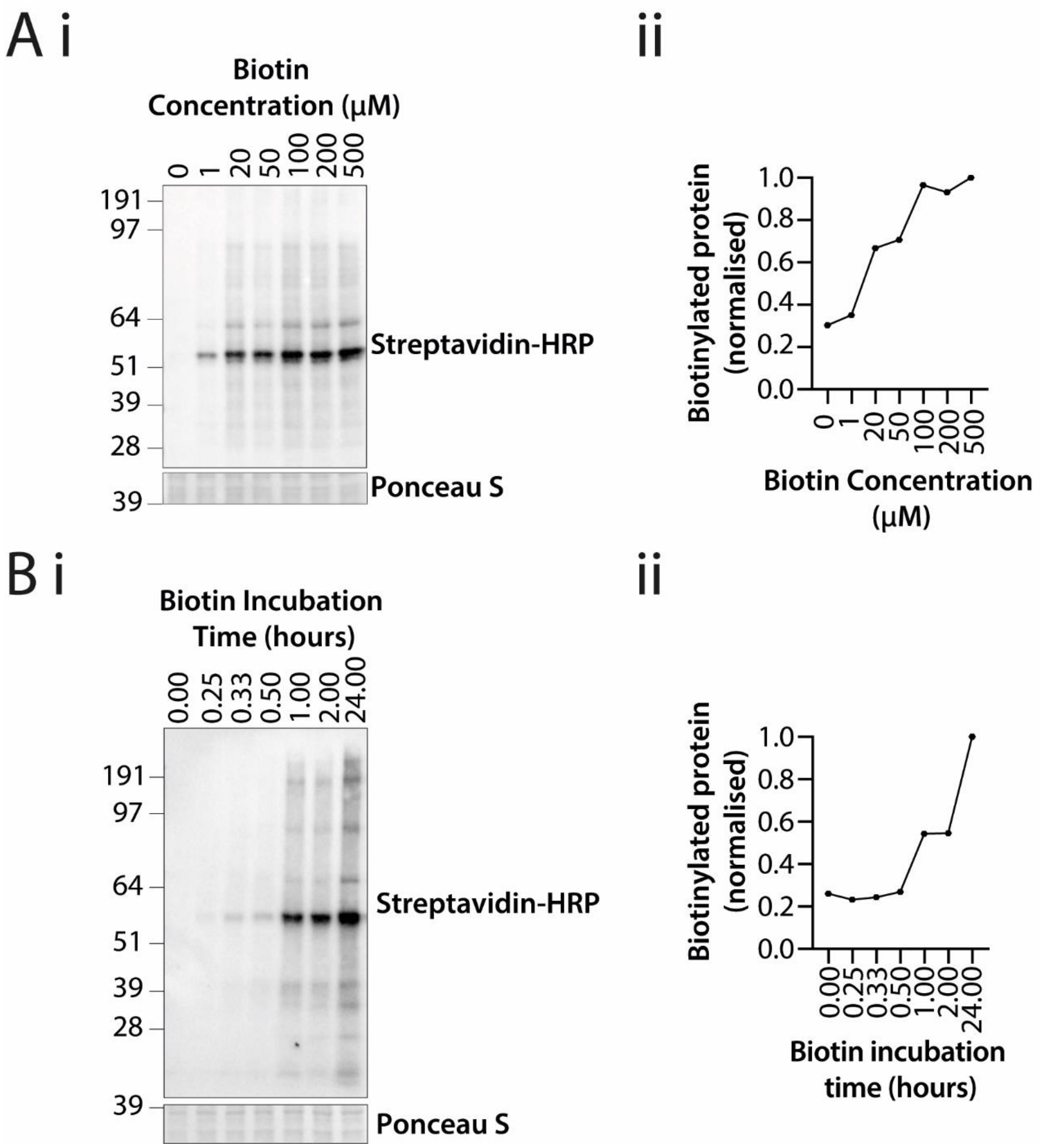

Supplementary Figure S1 Optimisation of miniTurbo biotinylation.

(A) Western blot of HEK293T cells transfected with WT CSP-V5-miniTurbo and subsequently incubated in 0-500 μM biotin for 24 hours (i), quantified by densitometry and normalised to total protein (Ponceau S) (ii) (n=1). (B) Western blot of HEK293T cells transfected with WT CSP-V5-miniTurbo and subsequently incubated with 50 μM biotin for 0-24 hours (i), quantified by densitometry and normalised to total protein (Ponceau S) (ii).

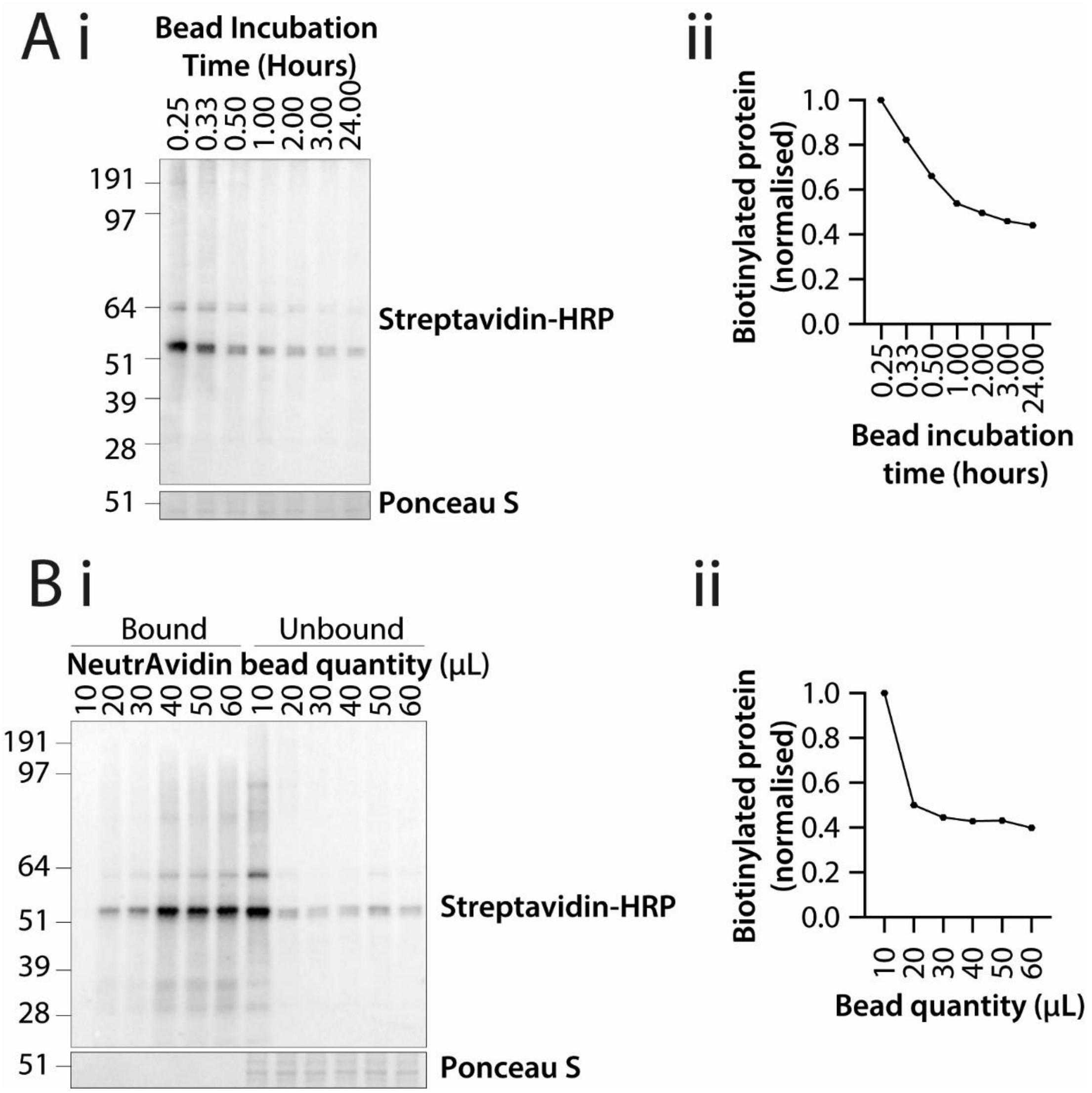

Supplementary Figure S2 Optimisation of biotinylated protein capture.

(A) Western blot of HEK293T cells transfected with WT CSP-V5-miniTurbo, incubated with 50 µM biotin for 24 hours, prior to biotin affinity purification. Unbound lysates were collected at various timepoints between 15 minutes and 24 hours and probed for streptavidin-HRP (i), quantified by densitometry and normalised to total protein (Ponceau S) (ii) (n=1). (B) Western blot of HEK293T cells transfected with WT CSP-V5-miniTurbo, and incubated with 50 µM biotin for 24 hours, prior to biotin affinity purification, using 10-60 µL Neutravidin beads and probed for streptavidin-HRP (i). Relative quantity of biotinylated protein in the unbound flow-through was quantified by densitometry and normalised to total protein (Ponceau S) (ii).

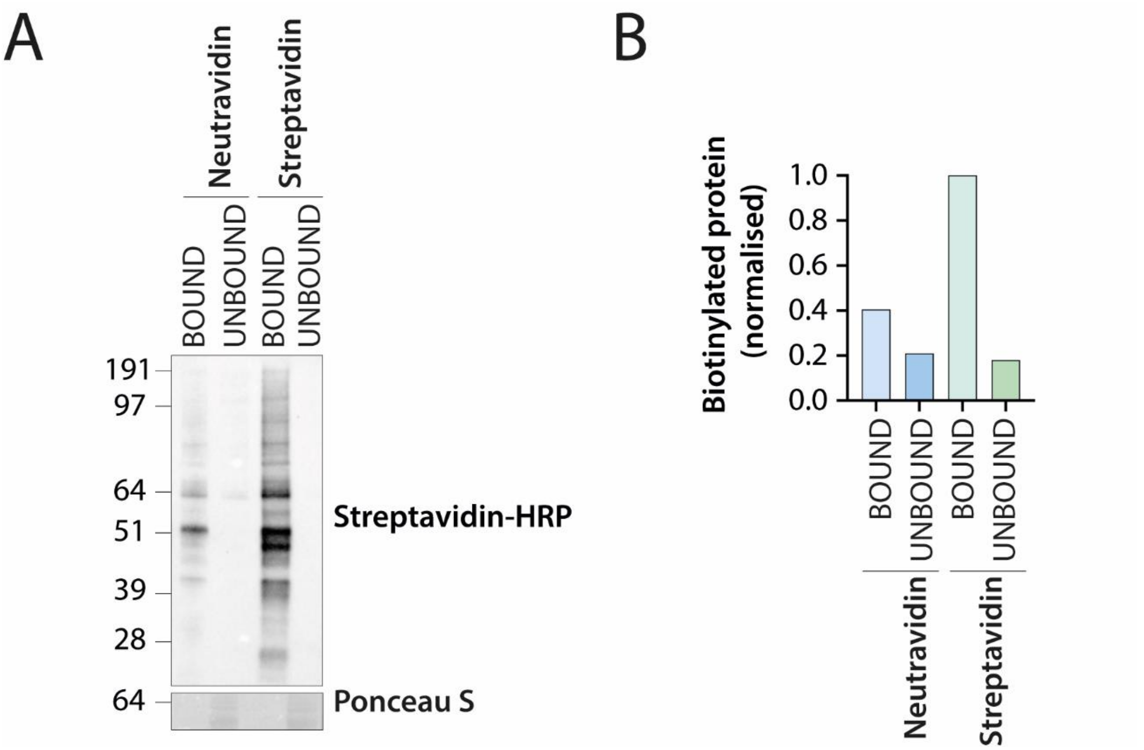

Supplementary Figure S3 Streptavidin beads capture increased quantities of biotinylated proteins.

(A) Western blot of HEK293T cells transfected with WT CSP-V5-miniTurbo, and incubated with 50 µM biotin for 24 hours, prior to biotin affinity purification, using neutravidin or streptavidin beads and probed for streptavidin-HRP. Samples loaded include proteins eluted from the beads (bound) and the flow-through (unbound). (B) Quantification of western blot in (A) through densitometry and normalisation to Ponceau S.

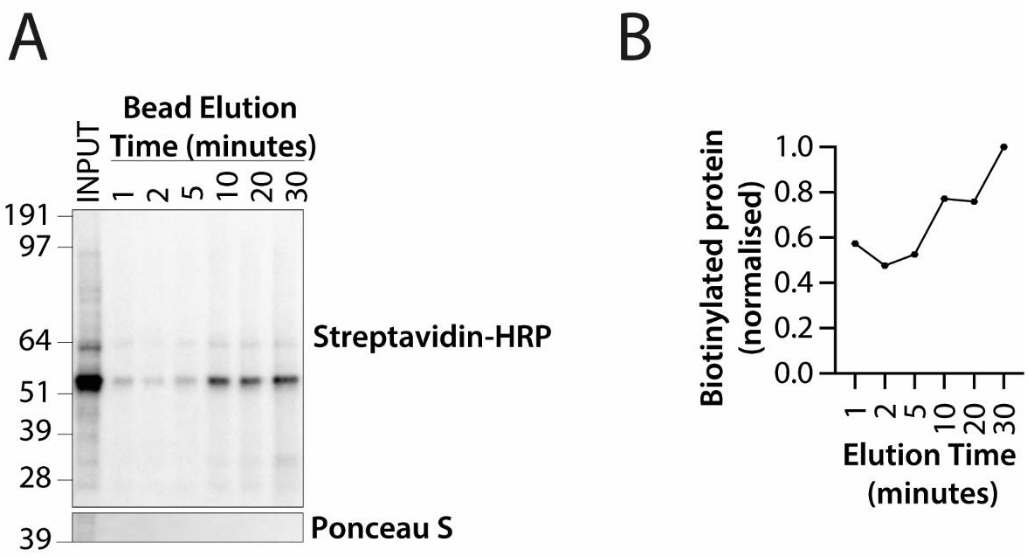

Supplementary Figure S4 Optimisation of biotinylated protein elution.

(A) Western blot of HEK293T cells transfected with WT CSP-V5-miniTurbo, incubated with 50 μM biotin for 24 hours, prior to biotin affinity purification and subsequent elution following 1-30 minutes boiling in SDS. Levels of biotinylated protein were assessed by western blotting using streptavidin-HRP. (B) Quantification of western blot in (A) by densitometry and normalised to total protein (Ponceau S).

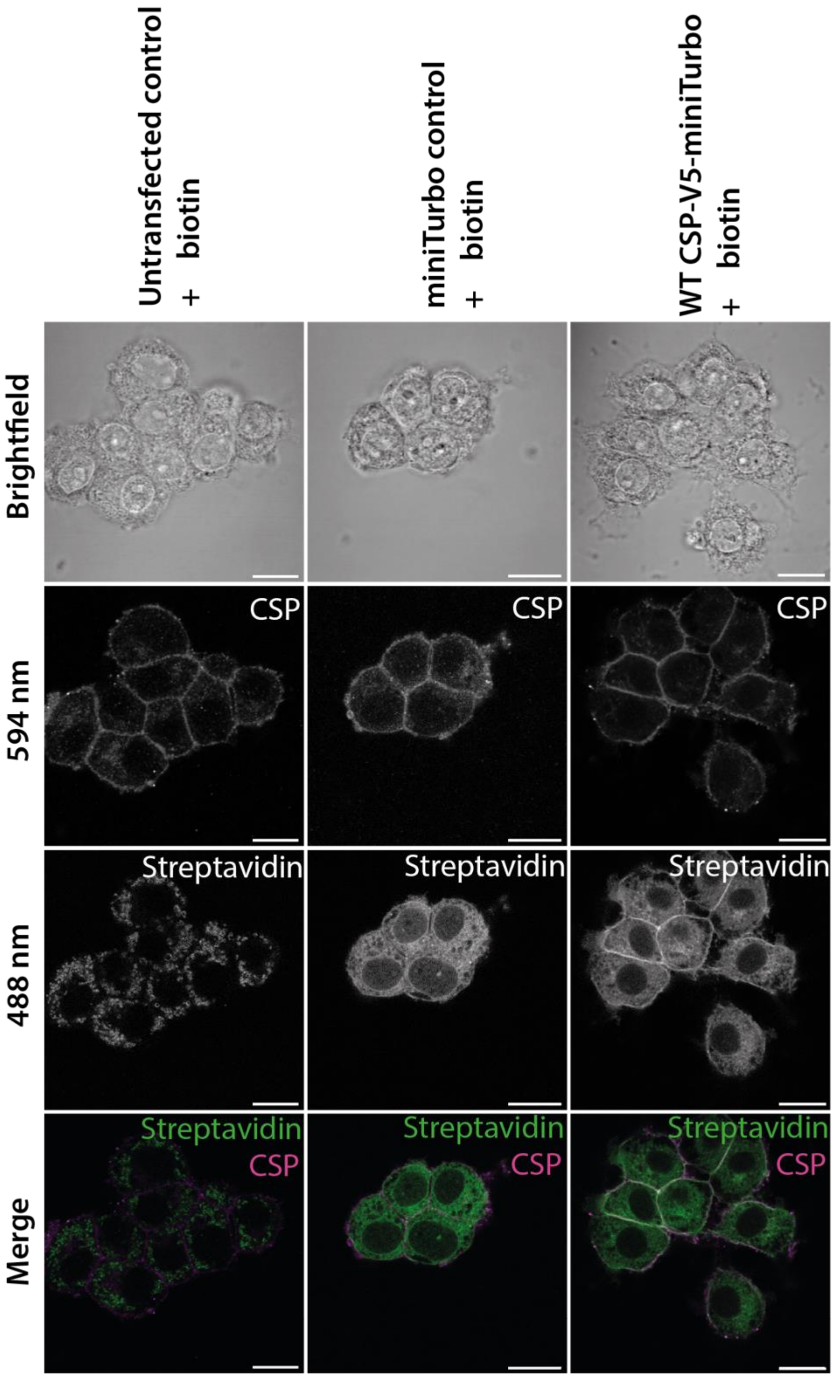

Supplementary Figure S5 Immunofluorescence of PC-12 cells stably transfected with miniTurbo control or WT CSP-V5-miniTurbo, with untransfected controls, in the presence of biotin and probed for CSP and streptavidin. Representative images shown were acquired on a Zeiss Axio Examiner ZI LSM880, using 488 nm and 594 nm excitation laser with 63× objective lens. Scale bars: 10 μm.

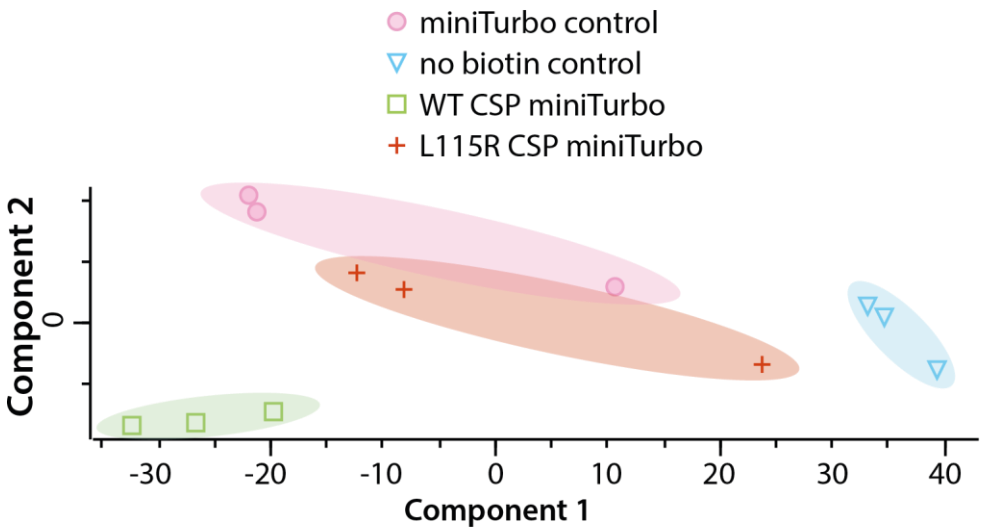

Supplementary Figure S6 Principal component analysis of LC-MS data.

Datasets compared include that from WT/L115R CSP-miniTurbo biotin affinity purification, including empty miniTurbo and no biotin controls. Each of the three independent biological replicates for each condition is indicated by the relevant symbols.
